# Using fluorescence fluctuation spectroscopy to quantify molecular dynamics within cells

**DOI:** 10.1002/2211-5463.70348

**Published:** 2026-10-08

**Authors:** Mark A. Hink

**Affiliations:** ^1^ van Leeuwenhoek Centre for Advanced Microscopy, Molecular Cytology, University of Amsterdam the Netherlands

**Keywords:** concentration, fluorescence correlation spectroscopy, intracellular measurements, mobility, number and brightness, oligomerization

## Abstract

Originally developed for the field of physical chemistry, fluorescence fluctuation spectroscopy (FFS) has evolved into a family of methods to quantify concentrations, diffusion rates and interactions of fluorescently labeled molecules. Combining microscopy with the ability to measure molecules at the nanomolar level allows for the study of biological processes with high sensitivity even within the living cell. In this protocol, the principles of how to set up these types of experiments are described including calibration samples, challenges and specific cellular applications. The predecessor to FFS, fluorescence correlation spectroscopy (FCS) will be discussed which measures diffusion and concentrations at a user‐defined point within the cell. Furthermore, an imaging variant, number and brightness analysis (N&B), will be shown that can be used to quantify molecular oligomerization. Overall, this protocol provides a foundation for using FFS and associated techniques to examine molecular dynamics within cells.

## Graphical abstract

Microscopy techniques like fluorescence correlation spectroscopy (FCS) and number and brightness analysis (N&B) allow to quantify concentrations, diffusion rates, and oligomerization of fluorescently labeled molecules in the living cell. In this protocol the principles of how to set up these types of experiments are described including calibration samples, challenges, and specific cellular applications.

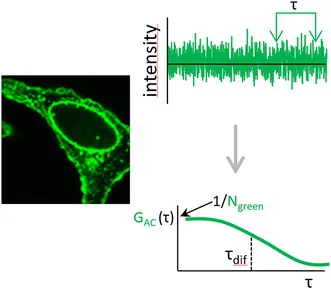

**Abbreviations**
APD: Avalanche Photo Diode
DMEM: Dulbecco's modified Eagle medium
DNA: Deoxyribonucleic acid
eGFP: Enhanced green fluorescent protein
FBS: Fetal bovine serum
FCS: Fluorescence correlation spectroscopy
FFS: Fluorescence fluctuation spectroscopy
FLIM: Fluorescence lifetime imaging microscopy
HeLa: Henrietta Lacks immortalized cell type
HyD: Hybrid detector
mTq2: Monomeric turquoise 2
N&B: Number and brightness analysis
NA: Numerical aperture
PBS: Phosphate‐buffered saline
PMT: Photo multiplier tube
PSF: Point spread function
sGFP2: super Green Fluorescent Protein 2

Fluorescence fluctuation spectroscopy (FFS) has evolved to a family of methods to quantify concentrations, diffusion rates, interactions, and oligomerization of fluorescently labeled molecules. In the following section, a protocol is presented to measure the concentration and mobility of fluorescently labeled proteins using Fluorescence Correlation Spectroscopy (FCS) [1] or the oligomerization state using Number and Brightness analysis (N&B) [2] inside living HeLa cells, utilizing an FCS‐optimized confocal microscope. The principle of these techniques is that at a low concentration fluorescently labeled molecules diffuse into and out of a small detection volume, like in a pixel as measured by a confocal microscope. The intensity fluctuations of typically 30–120 s measurements are either autocorrelated in time (FCS) or the signal statistics of each pixel in a stack of ca 50 images in time are calculated (N&B) (Fig. 1).

**Fig. 1:**
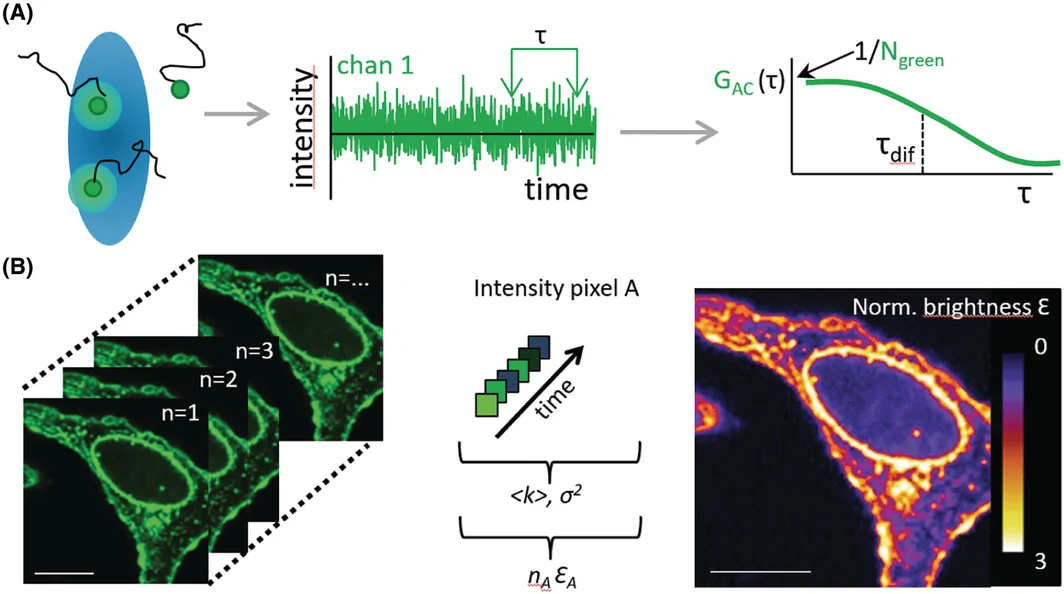
Principle of FCS (A) and N&B (B) techniques. At low concentrations, fluorescently labeled molecules diffuse into and out of the confocal microscope detection volume. The measured fluctuating intensity in time is autocorrelated resulting in the FCS curve that can be fitted to models to retrieve parameters like particle number and diffusion time. In N&B analysis, pixel intensities in a time‐stack of images are fluctuating. By calculating the average and variance of the intensity for each pixel, the number and brightness of the molecules can be estimated. The scalebar corresponds to 5 μm.

## Materials

1. Alexa 488 dye. This is obtained by letting the Alexa Fluor 488 NHS Ester (Succinimidyl Ester, Invitrogen, Waltham, MA, USA) react to water.
2. Phosphate‐buffered saline (PBS), pH 7.4.
3. Blackened glass‐bottomed 96‐well plate (Eppendorf, Enfield, CT, USA).
4. HeLa cell culture (ATCC, Manassas, VA, USA), (RRID:CVCL_0030). These cells have been authenticated and tested for mycoplasma annually.
5. Dulbecco's modified Eagle medium with Glutamax supplement (Gibco, Waltham, MA, USA).
6. ‘Transparent’ DMEM with low glucose, pyruvate, no glutamine & no phenol red (Gibco).
7. Fetal bovine serum (FBS) (Gibco).
8. Glutamax (Gibco).
9. T25 TC Cell culture flasks (Sarstedt, Nümbrecht, Germany).
10. Cell incubator at 7% CO_2_ 37 °C (Sanyo, San Diego, CA, USA).
11. Polystyrene 6 well cell culture plates (Costar, Corning Life Sciences, Amsterdam, the Netherlands).
12. Round glass cover slips of 24 mm diameter, glass thickness 1.5 (Epredia, Breda, the Netherlands).
13. HBBS salt medium (Gibco).
14. Trypsin (Gibco).
15. Lipofectamine 3000 (Invitrogen).
16. Opti‐Mem transfection medium (Gibco).
17. pUC18 DNA, empty plasmid used as carrier DNA (https://www.addgene.org/50004/).
18. DNA control plasmids encoding for monomeric and dimeric GFP. If higher order oligomeric complexes will be studied, also higher order control plasmids are preferred. Here the eGFP oligomer set created by the Kinjo lab will be used [3].
19. DNA plasmid encoding for Annexin‐A4‐sGFP2 [4].
20. Attofluor Cell Chamber ring (Invitrogen) to hold the round coverslips
21. Microscopy medium, 20 mm HEPES pH = 7.4, 137 mm NaCl, 5.4 mm KCl, 1.8 mm CaCl_2_, 0.8 mm MgCl_2_, 20 mm Glucose.
22. Ionomycin, Calcium Salt (Thermo Fisher, Waltham, MA, USA).
23. Olympus FV 1000 confocal microscope with 60× silicon oil immersed C‐apochromat objective lens with N.A. 1.4 (Olympus, Tokyo, Japan). The system is equipped with Picoquant (Berlin, Germany) pulsed diodes (including the LDH‐P‐C‐485 diode laser), controlled by a Sepia2 unit. The confocal microscope has a size‐flexible pinhole, multiple main dichroic mirrors, and multiple PMT detectors. However, the fluorescence is guided out of the microscope using an optical fiber (180 μm) into a custom‐made 4 APD (MPD, Bolzano, Italy) containing detection box (Picoquant). This box has flexible secondary dichroic mirrors and emission filters (Semrock, New York, NY, USA) that can be exchanged manually. The photons are registered by a Picoquant Picoharp300 unit. The microscope body is enclosed by an Okolab (Pozzuoli, Italy) incubator box such that the system can be heated to 37 °C for cellular measurements.
24. The confocal microscope is controlled by fluoview 4.0 software (Olympus) while the pulsed laser and APD detector are controlled by symphotime 64 software (Picoquant). In the latter software package, the FCS data analysis is carried out.
25. For N&B, the Picoquant arrival time data is converted into a tiff image stack using custom‐made software lcam ptu converter (http://www.lcam‐fnwi.nl/links/) and N&B images are generated and analyzed in simfcs4 (https://www.lfd.uci.edu/globals/).

## Methods

### Microscope and FCS calibration

1. The microscope is placed in an air‐conditioned room to ensure temperature stability and the calibration samples are stored in blackened glass‐bottomed 96‐well plates. In order to reduce the amount of detected background light, a protective blackened aluminum cover will be placed on top of the sample during the measurements.
2. Turn the microscope and lasers on roughly 30 min before starting the measurements in order to stabilize. When living cells will be measured in the same session, all measurements will be conducted at 37 °C using the Okolab incubator box.
3. Select the excitation laser line: The Alexa488 & GFP‐labeled samples will be excited with the 485 nm laser line, close to the absorbance maximum of the dyes. Tips & Tricks 1.
4. Select an appropriate objective lens. Since the critical value in FFS techniques is the detected molecular brightness, for example, the number of photons registered from a single molecule when passing the observation volume, a high numerical aperture (N.A.) lens should be selected. Here the 63× silicon oil immersed C‐apochromat lens N.A. 1.4 is chosen. Make sure the objective lens is clean. Tips & Tricks 2.
5. Configure the FCS microscope filters: The fluorescence is separated from the excitation light by a dichroic filter, reflecting at least the 485 nm excitation line (Chroma). The confocal pinhole is set at 1 Airy Unit. An appropriate bandpass filter that blocks the laser light and detects the main fluorescence band (e.g., BP500‐550) is used and placed in front of the APD detector. Tips & Tricks 3 & 4.
6. Measure the fluorescence of 200 μL of a ~ 100 nm solution of Alexa488 in phosphate‐buffered saline (PBS) in the 96‐well plate at a laser power of approximately 10–50 kW·cm^−2^. Focus into the sample using the sensitive APD detectors to distinguish Alexa signal from sample holder glass or silicon oil signal (when focused below the glass). Due to the low dye concentration in this solution, visual inspection or detection using PMT confocal detectors will not work. In order to prevent that one optimizes scattered excitation light or adsorbed dye from the bottom of the sample holder instead of observing the wished fluorescence signal, the focus should be set at least 20 μm into the solution, similar to the height where the measurements will take place in the living cell later. One should note that if one measures deeper into the sample, one could encounter issues when there is a refractive index difference between the sample and the objective immersion liquid that would affect the observation volume and therefore the quality of the measured parameters [5].
7. Adjust the correction collar of the objective lens to the position where the highest fluorescence count rate is observed. By adjusting this position, one corrects for the small optical aberration caused by the glass of the sample holder, disturbing the ideal PSF. Although most lenses have a glass thickness scale printed on the objective collar, the theoretical glass thickness value will often be close but not exactly the experimental optimal value.
8. If possible, optimize the detected fluorescence signal to its highest value by moving the X‐, Y‐, and Z‐position of the pinhole in the confocal microscope and optimize the lens position in front of the APD if possible.
9. Perform an FCS measurement of Alexa 488 using roughly 3 kW·cm^−2^ laser light. Adjust the measurement time such that the resulting correlation curve is smooth in the decaying part of the curve. For a well calibrated microscope, one should get a good quality curve within 30–120 s for this dye solution. Dependent on the detector type saturation should be prevented, which corresponds to an upper limit of ~ 500 kHz for the APDs used here.
10. Autocorrelate the intensity data and fit the resulting autocorrelation curve G(tau) (see section below).
11. One expects a diffusion time for Alexa488 in buffer of several tens of microseconds with a volume structure parameter between 4 and 10. Tips & Tricks 5.

### FCS data analysis

1. Process the data in the picoquant symphotime software. Select the part of the intensity trace that is stable in time, having maximally 10% bleaching, thus intensity decrease, per minute and autocorrelate this section using correlation time tau between 1 μs and 1 s. Moderate bleaching or other small drift in the signal could be corrected using high‐pass filtering or dividing the time trace into smaller segments and averaging the fit results over all individual autocorrelation curves.
2. Fit the resulting autocorrelation curve G(tau) to a fit model including terms for triplet‐state kinetics, three‐dimensional Brownian diffusion and an amplitude offset [6].
3. Judge the quality of the fit where the fitted curve data points should be through the center of the experimental data points also depicted by the fit residuals. These fit residuals should not show a wave pattern and should scatter randomly around zero. Tips & Tricks 6 (Fig. 2).

**Fig. 2:**
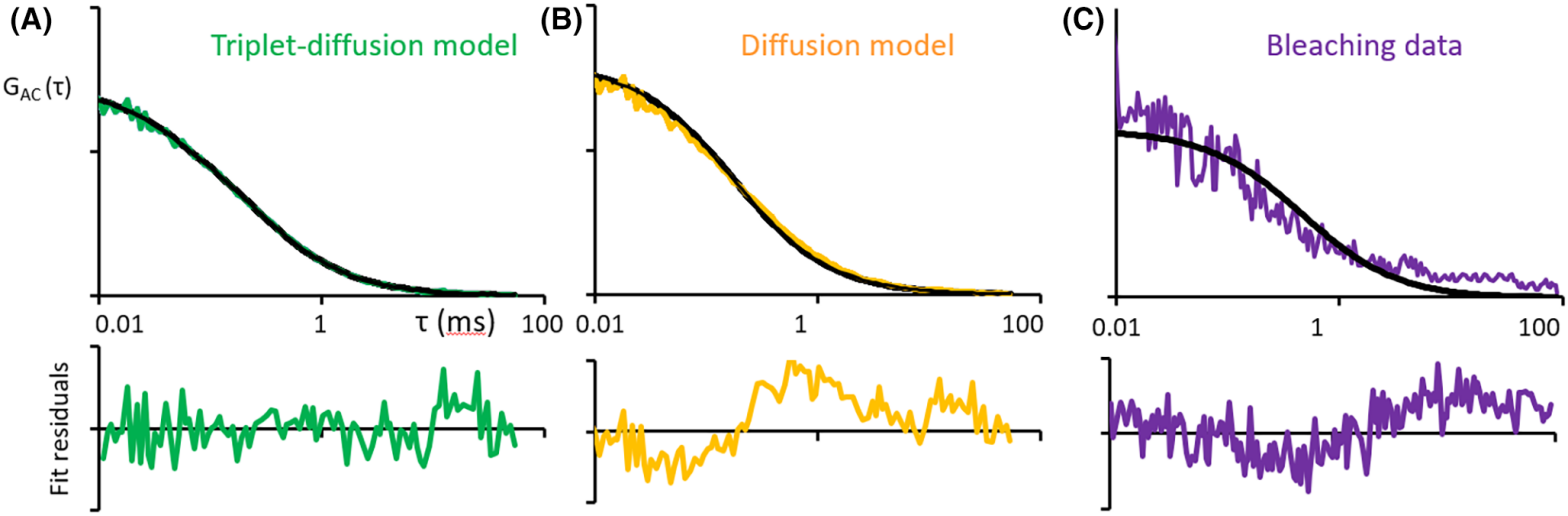
FCS data fitting. (A) The experimental decay curve derived from the measured intensity of nanomolar Alexa488 dye in PBS buffer (gray line) is fitted with a triplet‐1 component diffusion model (black line) resulting in small and almost shapeless fit residuals. However, when the triplet state is omitted from the model, a misfit can be seen (B), especially visible by the wave pattern in the fit residuals. (C) Significant photobleaching will result in a decrease of the measured intensity over time, a potential large amplitude decay at very high time scales in the correlation curve that could result in a misfit or obtaining the wrong fit parameters for diffusion time and particle number.

### FCS measurements in cells

1. HeLa cells are cultured in T25 culture flasks using standard DMEM medium supplied with 10% FBS. Two days before the experiment a flask full of cells is washed with HBBS medium, then trypsinized and ~ 20% flask volume is passed to a six‐well plate with each well containing a round coverslip of 24 mm diameter. The medium is replaced with transparent DMEM medium, to reduce autofluorescence, supplied with 10% FBS and 10% Glutamax.
2. The next day the cells are transfected. 10 ng of plasmid DNA encoding for the construct (e.g., Annexin‐A4‐sGFP2) is mixed with 90 ng carrier DNA and mixed with 2 μL Lipofectamin3000 and 50 mL Opti‐Mem. After 20 min, this mixture is added to the cells. Tips & Tricks 7.
3. The next day a coverslip is transferred from the incubator to the metal Attofluor ring and 1 mL of microscopy medium is added. The ring is placed on top of the microscope table where the whole system has been preheated to 37 °C.
4. Select a dim cell using the mercury lamp in combination with a GFP filter cube. A confocal image will be made using the internal PMTs and the laser beam is parked typically just besides the cell nucleus because of the large height of the cell cytoplasm for the typically hat‐shaped HeLa cells. Measure the fluorescence using the sensitive APD detectors at a laser power of approximately 1–5 kW·cm^−2^ for 1–2 min and lower the power when the fluorescence is dropping by more than 10% per minute.

### N&B calibration and measurements in cells

1. Select a dim cell at the glass coverslip with control cells transfected with the monomeric GFP construct using the mercury lamp and/or the confocal microscope.
2. Take a time series of 50 images, typically with a limited number of pixels (e.g., 128 × 128 or 256 × 256 pixels) and choose a pixel size close to the size of the confocal Point Spread Function (PSF) (250–500 nm). In such an image, two to three HeLa cells could be measured.
3. Set the two most critical experimental parameters: The laser power is set at approximately 1–5 kW·cm^−2^ preventing photobleaching more than 10% per minute and the pixel dwell time is close to 100 μs. Tips & Tricks 8
4. Repeat the measurement for the cells transfected with the dimer‐GFP construct.
5. Analyze the images (see Fig. 3) and compare the brightness values for the monomeric and dimeric GFP control samples.
6. Optimize the laser power and pixel dwell time such that a clear difference in brightness between the monomeric and dimeric GFP samples could be obtained. If the standard deviation of the obtained brightness value is high, one could choose a higher laser power and pixel dwell time and/or collect more than 50 images.
7. The Annexin‐A4‐sGFP2 protein will translocate from the monomeric form in the cytoplasm to the cell plasma membrane and starts to oligomerize from a monomer via a trimeric intermediate form into a high order aggregate within a few minutes after inducing the cells with 1 μg·mL^−1^ ionomycin [4].

**Fig. 3:**
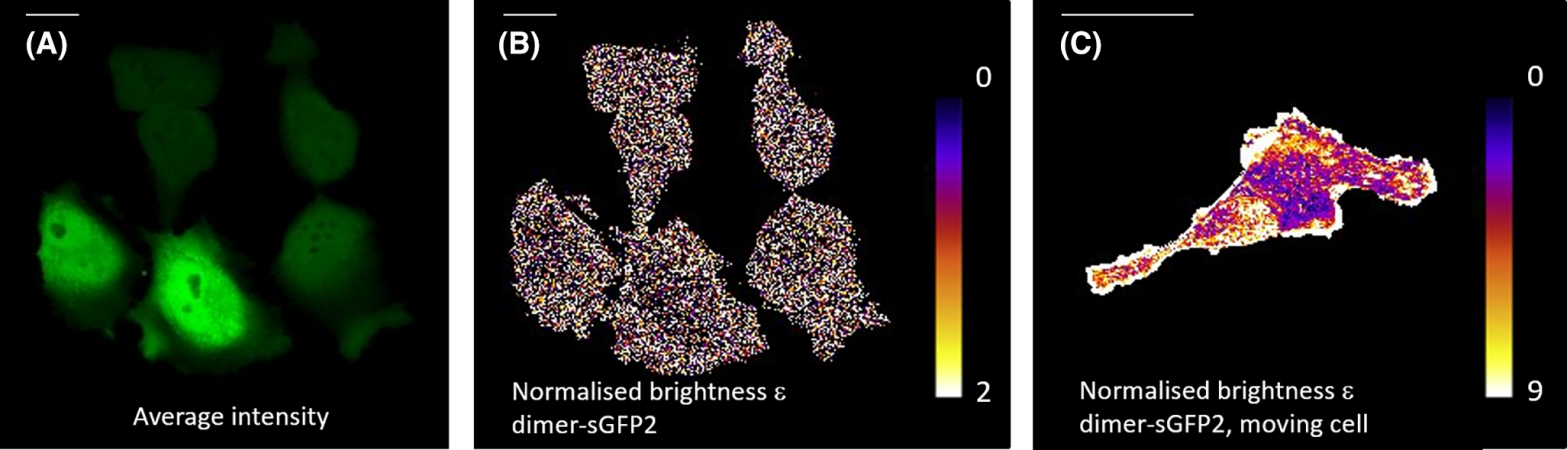
N&B data fitting. From the image stack of HeLa cells transfected with DNA plasmid encoding for monomeric sGFP2 (A) the brightness image is created (B) by dividing the intensity variance by the average for each pixel. For moving cells, an artificial high brightness will be obtained in the pixels at the edges of the cell (C). The scalebar corresponds to 5 μm.

### N&B data analysis

1. If photobleaching or small cellular drift is present, this can be corrected for in the software.
2. Cells should not move during the time sequence since these intensity fluctuations can cause extremely high brightness values esp. in pixels close to the edges of the cell or close to dark organelles. Cells or regions of cells that display obvious movement artifacts are excluded from the analysis.
3. Molecular brightness values in the sample could be normalized to the monomeric and dimeric calibration samples. For the sGFP2 dye selected here, the maturation rate and efficiency is such high (99%) that hardly any dark FP protein is present in the lower oligomeric forms. However, for many other FPs a correction for nonfluorescent proteins would be required [7]. This together with potential microscope polarization effects typically results in a slightly lower brightness of the dimer than the expected doubling of the monomer brightness. For a heterogenous distribution of multiple oligomeric states, a weighted average brightness would be obtained that does not scale only to the molecular fraction but also to the squared relative brightness of each species similar to [8].

## Tips & Tricks/troubleshooting

1. For the intracellular measurements, we use fluorescent proteins that are fused genetically to the proteins of interest. Key parameter in FFS experiments is the molecular brightness of the dye which is the number of detected photons per molecule per time unit. This value correlates to the product of the molecular extinction coefficient multiplied with the quantum yield of the dye. These values can be obtained from FPbase (www.fpbase.org) or Fluorofinder (https://fluorofinder.com) for chemical dyes. However also other factors play a role here since most dyes are not fluorescing constantly: Dyes could be sensitive to photobleaching or have a large fraction of dark‐state that could be related to dye blinking, caused for example by (laser power dependent) cis‐trans isomerization of the chemical dye structure [9] or a limited maturation rate of the FP since these proteins require, sometimes slow, single or double oxidation steps within the cell before the freshly synthesized FP will become fluorescent [10]. Many of the Atto & Alexa chemical dyes and FPs like mTq2, eGFP & mScarlet3 are very well suited for FFS applications.
2. Besides selecting the most bright fluorescent dye (see Tips & Tricks 1), also the detection path should be optimized. Therefore, it is crucial to select an objective lens with a high numerical aperture that allows for photon detection at wide angles and detectors with high detection efficiency. In addition, the FCS data fitting models assume to have a well‐defined (typical) Gaussian shaped observation volume. To approximate this shape as best as possible, the immersion medium of the objective lens should be as close to the refractive index of the sample as possible. Thus, for buffer measurements, a water immersed lens would be the best choice. If available, one could select a silicon immersed lens (refractive index silicon oil of 1.40). This is even closer to the typical intracellular refractive index of approximately 1.38 than using a water immersed lens (refractive index 1.33) [11]. A too high laser power could saturate the dye (and/or detector), which will deform the apparent detection volume [12].
3. Although here a pulsed laser unit has been used, pulsing at a frequency of 20 MHz, this is not essential for the experiment and continuous wave lasers could be used as well. However, the advantage of a pulsed laser would be that one could acquire fluorescence lifetime (FLIM) information simultaneously with the FCS or N&B experiment. This is especially useful to distinguish the very low expressing transfected cells from the nontransfected wild‐type cells since the FLIM pattern of eGFP/sGFP2 will give an almost straight decay profile (in the typical semi‐log space FLIM graph) while autofluorescence will result in an almost exponential‐shaped and fast decaying pattern. The FLIM information would also allow to perform lifetime‐FCS where one can distinguish multiple dyes not on the basis of color but on the basis of different lifetime properties [13].
4. Since FFS analyses the molecular noise of diffusing and blinking molecules, the detectors used should not create additional detection or read‐out noise. Therefore, classical photomultiplier tubes (PMT) will not be used; instead, photon‐counting‐based detectors like avalanche photodiodes (APD) or hybrid detectors (HyD) are preferred.
5. If data are noisy in the beginning of the curve, a part of the G(tau) curve can be excluded from fitting as long as the important items for the researcher (amplitude for concentration estimation and/or decay part for mobility values) are included in the fit range.
6. A wave pattern in the fit residuals indicates that the selected fit model is not describing the system completely, for example, it might be that inside the cell a molecule shows two instead of one diffusional behavior like free diffusion plus slow diffusion when the molecule is complexed with a larger intracellular binding partner or when a measurement spot is used in a region where anisotropic diffusion is taking place, like cytoplasmic diffusion between the plates of the endoplasmatic reticulum. In this case, the diffusional fit model can be extended using 2 instead of 1 diffusion terms. However, for the calibration measurement only a single diffusion term is expected to be valid.
7. FFS experiments should be performed at the nanomolar concentration range of dye. At lower concentrations, one measures less than one dye molecule per detection volume; thus, one would require very long measurement times for a statistically good and trustworthy dataset (scanning techniques are possible to overcome this issue). When the dye concentration largely exceeds the 10 μm level, typically used for standard confocal imaging, the fluorescence intensity fluctuations of the many dye molecules start to cancel out each other and the FFS technique will not work properly. In order to achieve correct concentration levels within the transfected cell, one can vary the DNA construct and the transfection protocol. When the DNA construct contains a very strong promoter sequence (like viral CMV or plant P35 sequence), one has to transfect low amounts of DNA and/or reduce the time between transfection and microscopy measurement. Note that many transfection protocols require a minimal amount of DNA for an efficient transfection. For transfections with a low amount of the plasmid of interest, one could add extra noncoding (carrier) plasmid vector to reach the minimum required total amount of DNA. Alternatively, one could create DNA constructs with low expression or inducible promoter sequences.
8. For Number & Brightness measurements, a critical experimental setting is the pixel dwell time. A too large pixel dwell time will cause that the detected pixel signal is constant due to the averaging effect of detecting many fluorescent dyes passing by in the detection pixel. On the other hand, a too small pixel dwell time results in a very low number of detected photons, thus poor statistics. The ideal pixel time is close to 10–50% of the average diffusion time of the dye, that could be estimated from a FCS experiment and in practice will be typically between 100 and 1000 μs. Note that the selected pixel dwell time should not be changed between the different samples. In order to increase the quality of the data, one could increase the laser power in order to detect a higher number of photons until one will generate too much photobleaching artifacts, observed when the detected signal is decreasing more than 15% per minute. Control constructs consisting of at least monomeric and dimeric (and if wished higher order oligomeric) dyes are measured to validate that the microscope settings will allow for the discrimination between these different oligomeric samples. If the detected intensity is too high for the detector, deadtime artifacts could lower the estimated brightness.

## Conflict of interest

The author declares no conflict of interest.

## Author contributions

MAH conceived and designed the project, acquired the data, analyzed and interpreted the data, and wrote the paper.

## Data Availability

Plasmids encoding the different intracellular signals or FP tags are available for nonprofit research organizations via the nonprofit repository Addgene (www.addgene.org/Dorus_Gadella). Data are available via www.lcam‐fnwi.nl/data.
